# The risk factors and predictive nomogram for postoperative hypoalbuminemia in elderly patients undergoing hip arthroplasty

**DOI:** 10.3389/fnut.2026.1912982

**Published:** 2026-07-31

**Authors:** Zhengyou Hou, Yiran Liu, Jingyuan Zhang, Bingbing Xiang

**Affiliations:** 1 Department of Anesthesiology, Chengdu Fifth People’s Hospital (The Second Clinical Medical College, Affiliated Fifth People’s Hospital of Chengdu University of Traditional Chinese Medicine), Chengdu, China; 2 School of Public Health, Anhui University of Science and Technology, Hefei, China; 3 Department of Anesthesiology, Second Affiliated Hospital of Kunming Medical University, Kunming, China

**Keywords:** clinical nutrition, elderly patients, hip arthroplasty, postoperative hypoalbuminemia, prediction model, risk factors

## Abstract

**Background:**

Postoperative hypoalbuminemia is a common nutritional-related complication in elderly patients undergoing hip replacement surgery, which is a common nutritional-related disorder that reflects nutritional imbalance during the perioperative period and is closely associated with adverse clinical outcomes, such as an increased risk of postoperative infection and prolonged hospital stay.

**Objective:**

This study aimed to identify the independent risk factors for postoperative hypoalbuminemia in elderly patients undergoing hip arthroplasty, and to develop a nomogram-based risk prediction model for perioperative clinical application. The ultimate goal was to facilitate early identification of high-risk patients and guide targeted interventions to reduce postoperative complication rates.

**Methods:**

A retrospective cohort study was conducted, enrolling 308 elderly patients who underwent hip arthroplasty from January 2015 to September 2024. Univariate analysis was first performed to screen potential risk factors. Variables with a *p* value <0.05 in univariate analysis were then included in multivariate stepwise logistic regression to identify independent predictors. A nomogram was constructed to visualize the prediction model. Model performance was assessed using three metrics: discrimination (via area under the receiver operating characteristic [ROC] curve, AUC), calibration (via Hosmer-Lemeshow test and Brier score), and clinical utility (via decision curve analysis [DCA]).

**Results:**

Postoperative hypoalbuminemia occurred in 170 of 308 patients (55.19%). Multivariate regression identified five independent risk factors: total fluid intake, preoperative serum total protein (TP), hydroxybutyrate dehydrogenase (HBDH), international normalized ratio (INR), and plasma D-dimer. Model calibration was excellent: the Hosmer-Lemeshow test yielded a *χ*^2^ value of 6.997 (*p* = 0.537); the ROC curve yielded an AUC of 0.752 (95% CI: 0.699–0.805). The calibration curve showed good consistency between the predicted and actual probabilities. Decision curve analysis (DCA) showed that using the nomogram had a high net benefit.

**Conclusion:**

Total fluid intake, preoperative TP, HBDH, INR and D-dimer level are five independent risk factors for postoperative hypoalbuminemia in elderly patients undergoing hip arthroplasty. The nomogram-based prediction model developed in this study exhibits good calibration, good discrimination and favorable clinical utility, making it a practical tool for guiding targeted clinical nutritional interventions, thereby optimizing patients’ nutritional status and improving their clinical outcomes.

## Introduction

The global aging population has led to a significant increase in age-related musculoskeletal disorders, among which femoral neck avascular necrosis, femoral neck fractures, and hip osteoarthritis have become major health issues affecting elderly individuals (1, 2). Artificial hip replacement is the primary treatment for these end-stage hip diseases, effectively relieving pain and restoring hip function (3–5). However, elderly patients undergoing hip arthroplasty often have concurrent nutritional disorders, making clinical nutrition—an essential component of perioperative management—critical. Clinical nutrition aims to optimize patients’ nutritional status through scientific assessment and targeted intervention, thereby reducing postoperative complications and promoting recovery.

Hypoalbuminemia is one of the most common nutrition-related complications in these patients (6). Its occurrence is closely related to nutritional metabolism, mainly caused by insufficient intake of energy and essential amino acids (the material basis for albumin synthesis), impaired hepatic synthetic function, stress-induced enhanced catabolism, or abnormal albumin distribution (7). Importantly, hypoalbuminemia is not only a direct marker of nutritional insufficiency but also a key mediator of adverse clinical outcomes, including delayed wound healing, increased risks of surgical site infection and pneumonia, and prolonged hospital stay (8), which imposes significant economic burdens on patients and strains healthcare systems (9).

Despite its great clinical and nutritional significance, existing research on its risk factors in elderly patients undergoing hip arthroplasty is limited, and there is no validated nomogram specifically for predicting this complication. To fill this gap, this retrospective study (2015–2024) analyzed clinical data of elderly hip arthroplasty patients, aiming to identify independent risk factors and develop a nomogram prediction model, providing an evidence-based tool for perioperative nutritional management to improve patient outcomes.

## Materials and methods

This study protocol was approved by the Institutional Ethics Committee of the Second Affiliated Hospital of Kunming Medical University (Kunming, China, ID: PJ-2021-39). Clinical trial number: not applicable. Given the retrospective nature of the study design, the ethics committee waived the requirements for informed consent and clinical trial registration. All patient data were collected and analyzed anonymously, with no potential harm to participants. The study was conducted in strict accordance with the principles of the Declaration of Helsinki (revised 2013).

A single-center, retrospective cohort study was conducted. We enrolled elderly patients who underwent hip arthroplasty in the Department of Orthopedics between January 2015 and September 2024. Inclusion criteria: (1) Age ≥75 years at the time of surgery; (2) Underwent primary hip arthroplasty for pathological conditions including femoral neck avascular necrosis, femoral neck fracture, or hip osteoarthritis; (3) Complete medical records available for review (including preoperative and postoperative laboratory tests, surgical records, and postoperative follow-up data within 30 days of surgery). Exclusion criteria: (1) History of complex surgical procedures (e.g., combined orthopedic and abdominal surgery) during the same hospitalization; (2) Incomplete clinical data (e.g., missing preoperative serum albumin levels or postoperative outcome records); (3) Preoperative serum albumin level <30 g/L (to exclude patients with pre-existing severe hypoalbuminemia, which could confound the assessment of postoperative hypoalbuminemia); (4) Presence of acute infections (e.g., sepsis), liver cirrhosis, or end-stage renal disease (glomerular filtration rate <30 mL/min/1.73 m^2^) preoperatively (conditions known to independently affect serum albumin synthesis or metabolism).

**Figure 1:**
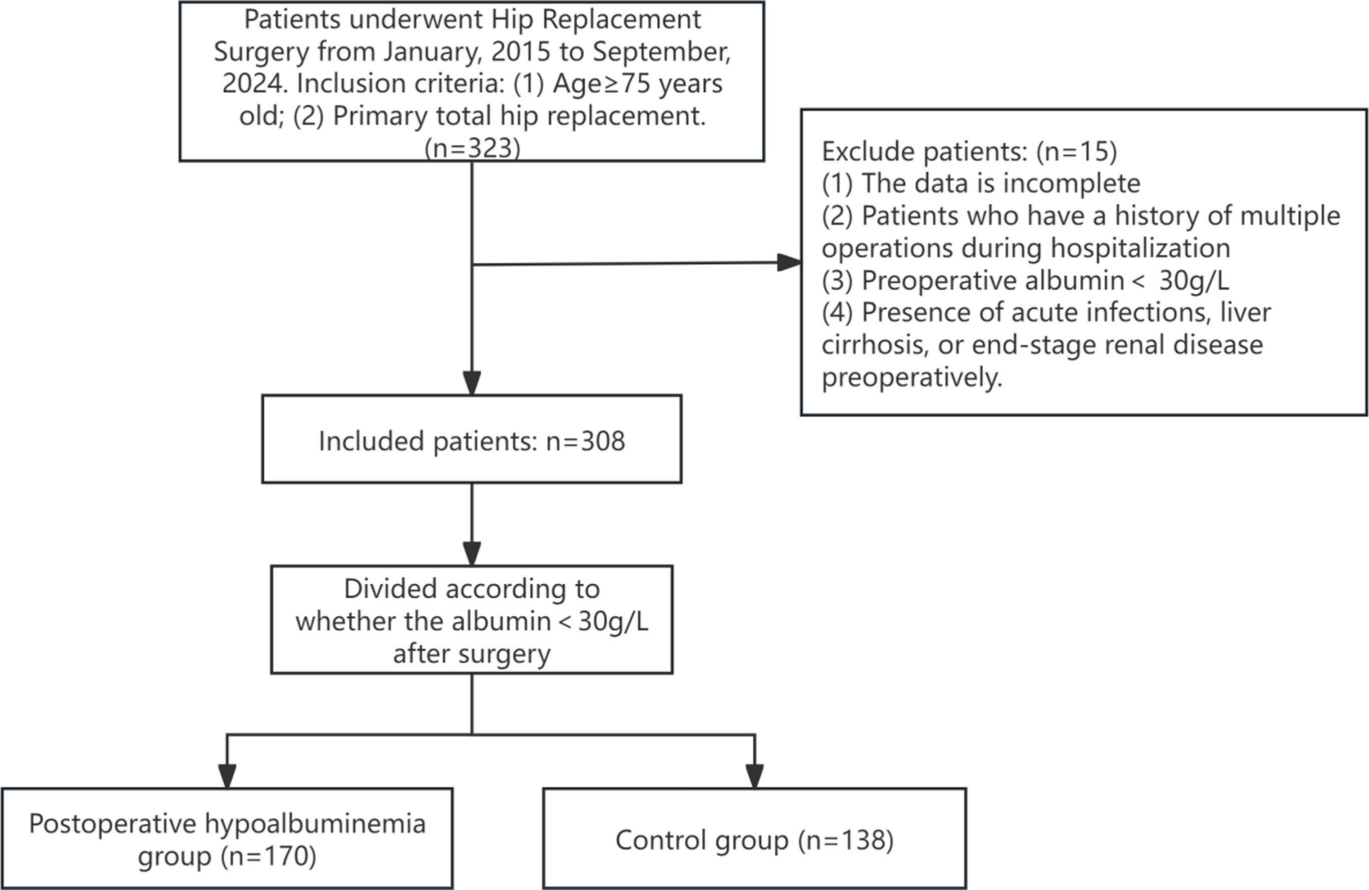
Flow chart of patients’ enrollment in this study.

A total of 323 patients were initially screened. After applying the exclusion criteria (3 patients with complex surgical history, 5 with incomplete data, and 7 with preoperative albumin <30 g/L), 308 patients were finally included in the analysis. The patient selection process is illustrated in a flow diagram (Figure 1). For the purposes of this study, clinically significant hypoalbuminemia was defined as serum albumin < 30 g/L. This threshold is consistent with current clinical guidelines, which recommend human albumin supplementation for patients with albumin concentrations <30 g/L. (6, 9) Therefore, in this study, subjects with the lowest postoperative albumin level <30 g/L were classified as hypoalbuminemia group, and the remaining subjects were included in the control group (10, 11).

All variables were extracted from the hospital’s electronic medical records system by two independent researchers to ensure data accuracy. Variables were categorized as follows: Demographic characteristics: Age (years), sex (male/female), height (cm), weight (kg), and body mass index (BMI, calculated as weight/height^2^, kg/m^2^). Comorbidity and preoperative status: Presence of hypertension (defined as systolic blood pressure ≥140 mmHg or diastolic blood pressure ≥90 mmHg, or use of antihypertensive medications), diabetes mellitus, cardiovascular disease (including coronary heart disease or heart failure), previous fracture history (yes/no), and American Society of Anesthesiologists (ASA) physical status classification (I–IV). Perioperative parameters: Anesthesia type, operation time (minutes, from skin incision to skin closure), Intraoperative total fluid intake (including crystalloids, colloids, and transfused blood products) and intraoperative blood loss. Laboratory indicators: Preoperative laboratory results (collected on the first day of admission) and postoperative laboratory results (collected on the day of surgery completion) including serum albumin (g/L), total protein (TP, g/L), hydroxybutyrate dehydrogenase (HBDH, U/L), international normalized ratio (INR), D-dimer (mg/L FEU), etc. Postoperative outcomes: Postoperative hospital stay (days), length of total hospital stay (days), and discharge status. All short-term adverse events, including postoperative pneumonia, acute kidney injury, ICU admission and all-cause mortality, were assessed within a 28-day postoperative surveillance window. Postoperative ICU admission was defined as any transfer to the intensive care unit occurring from the end of surgery up to postoperative day 28.

All statistical analyses were performed using R software (Version 4.3.1; R Foundation for Statistical Computing, Vienna, Austria) with the “rms” (Regression Modeling Strategies), “pROC” (Receiver Operating Characteristic Analysis), and “calibrate” packages. A two-tailed *p* value <0.05 was considered statistically significant. Continuous variables: Normally distributed variables (tested via the Shapiro–Wilk test) were presented as mean ± standard deviation (M ± SD) and compared between groups using the independent samples t-test. Non-normally distributed variables were presented as median and interquartile range [M (Q₁, Q₃)] (where Q₁ = 25th percentile, Q₃ = 75th percentile) and compared using the Mann–Whitney U test. Categorical variables: Presented as counts and percentages [N (%)] and compared between groups using the chi-square test, chi-square test with continuity correction, or Fisher’s exact test. All hypothesis tests were two-tailed, with a significance level set at *p* < 0.05.

Univariate logistic regression: All variables were first included in univariate logistic regression analysis to screen for potential risk factors associated with postoperative hypoalbuminemia. Multivariate logistic regression: A stepwise forward selection method was used to identify independent risk factors for postoperative hypoalbuminemia. The final multivariate logistic regression model was constructed using the identified independent risk factors, with the predicted probability of postoperative hypoalbuminemia calculated via the formula.

Nomogram construction: The final multivariate model was visualized as a nomogram using the “lrm” (Logistic Regression Model) function in the “rms” package. Each independent variable was assigned a corresponding score on the nomogram, and the total score of an individual patient was used to estimate the probability of postoperative hypoalbuminemia (with scores quantified to ensure clinical interpretability). The performance of the model is evaluated by plotting the area under the ROC curve (AUC), calibration curves and decision curve analysis (DCA). The line plot is visualized using actual results, and the impact of each variable on the prediction target is quantified to calculate the final prediction probability.

## Results

### Baseline characteristics of study participants

A total of 308 elderly patients who underwent hip arthroplasty were included in the final analysis. Among these, 170 patients (55.19%, 170/308) developed postoperative hypoalbuminemia (hypoalbuminemia group), and the remaining 138 patients (44.81%, 138/308) were classified into the control group. The median (interquartile range, IQR) age was 83.0 (80.0, 87.0) years in the hypoalbuminemia group and 81.0 (78.0, 85.0) years in the control group. Regarding sex distribution, 112 patients (65.9%) in the hypoalbuminemia group were female and 58 (34.1%) were male, whereas the control group included 93 female patients (67.4%) and 45 male patients (32.6%) (Table 1).

**Table 1:** Baseline characteristics and univariate analysis.

Variables	Control group (*n* = 138)	hypoalbuminemia group (*n* = 170)	*p*
Age(yr)	81.0 (78.0, 85.0)	83.0 (79.0, 85.0)	0.027
Gender			0.875
Female	93 (67.4%)	112 (65.9%)	
Male	45 (32.6%)	58 (34.1%)	
Weight(kg)	53.0 (48.0, 60.0)	53.0 (50.0, 60.0)	0.814
Height(m)	1.6 (1.5, 1.6)	1.6 (1.5, 1.6)	0.289
BMI(kg/m2)	21.7 (19.5, 23.4)	21.4 (19.5, 23.4)	0.806
Hypertension			0.26
No	56 (40.6%)	81 (47.6%)	
Yes	82 (59.4%)	89 (52.4%)	
Diabetes			0.548
No	109 (79%)	140 (82.4%)	
Yes	29 (21%)	30 (17.6%)	
Cerebral infarction			0.822
No	123 (89.1%)	149 (87.6%)	
Yes	15 (10.9%)	21 (12.4%)	
Coronary heart disease			1
No	123 (89.1%)	152 (89.4%)	
Yes	15 (10.9%)	18 (10.6%)	
Diagnosis			0.382
Avascular necrosis	29 (21%)	28 (16.5%)	
Fmoral neck fractures	109 (79%)	142 (83.5%)	
ASA			0.915
<3	64 (46.4%)	81 (47.6%)	
≥3	74 (53.6%)	89 (52.4%)	
ECG			0.515
Normal	101 (73.2%)	131 (77.1%)	
Abnormal	37 (26.8%)	39 (22.9%)	
Chest X ray			0.223
Normal	93 (67.4%)	102 (60%)	
Abnormal	45 (32.6%)	68 (40%)	
Preoperative anticoagulation			0.212
No	44 (31.9%)	67 (39.4%)	
Yes	94 (68.1%)	103 (60.6%)	
Creatine kinase-MB			0.45
≤25 U/L	134 (97.1%)	161 (94.7%)	
>25 U/L	4 (2.9%)	9 (5.3%)	
Preoperative anemia			0.013
No	121 (87.7%)	129 (75.9%)	
Yes	17 (12.3%)	41 (24.1%)	
Myoglobin			0.628
Male≤72 ng/mL Female≤58 ng/mL	122 (88.4%)	146 (85.9%)	
Male>72 ng/mL Female>58 ng/mL	16 (11.6%)	24 (14.1%)	
Brain natriuretic peptide (pg/mL)			0.058
≤100 ng/L	80 (58%)	79 (46.5%)	
>100 ng/L	58 (42%)	91 (53.5%)	
C-reactive protein			0.775
≤10 mg/L	116 (84.1%)	146 (85.9%)	
>10 mg/L	22 (15.9%)	24 (14.1%)	
Deep venous thrombosis			0.17
No	129 (93.5%)	150 (88.2%)	
Yes	9 (6.5%)	20 (11.8%)	
Total protein (g/L)	65.3 (61.7, 69.4)	62.0 (57.9, 68.0)	<0.001
Alanine transaminase (U/L)	14.0 (11.0, 23.0)	16.0 (11.0, 21.0)	0.939
Aspartate aminotransferase (U/L)	21.0 (17.0, 25.0)	22.0 (18.0, 27.0)	0.188
Total bilirubin (umol/L)	15.6 (11.4, 21.0)	16.7 (11.7, 21.9)	0.305
Direct bilirubin (umol/L)	5.9 (3.8, 7.3)	5.8 (3.9, 7.8)	0.416
Indirect bilirubin (umol/L)	10.2 (7.6, 13.2)	10.6 (7.6, 14.7)	0.271
Lactate dehydrogenase (U/L)	214.5 (181.0, 247.0)	212.0 (188.0, 256.0)	0.267
Hydroxybutyrate dehydrogenase (U/L)	145.5 (119.0, 166.0)	149.0 (131.0, 178.0)	0.036
Creatine kinase (U/L)	87.5 (63.0, 141.0)	107.0 (75.0, 175.0)	0.018
Urea nitrogen (mmol/L)	6.3 (5.2, 7.7)	6.6 (5.2, 7.7)	0.283
Creatinine (umol/L)	70.5 (58.0, 87.0)	73.0 (61.0, 90.0)	0.278
Uric acid (umol/L)	313.0 (233.0, 391.0)	312.5 (255.0, 379.0)	0.804
Triglyceride (mmol/L)	1.1 (0.8, 1.6)	1.0 (0.8, 1.4)	0.038
High density lipoprotein cholesterol (mmol/L)	1.2 (1.0, 1.5)	1.2 (1.0, 1.5)	0.839
Low density lipoprotein cholesterol (mmol/L)	2.6 ± 0.9	2.4 ± 0.8	0.063
Blood glucose (mmol/L)	5.6 (5.0, 6.8)	5.6 (5.0, 6.5)	0.712
Potassium (mmol/L)	3.8 ± 0.5	3.9 ± 0.5	0.367
Chloridion (mmol/L)	105.4 (103.0, 107.2)	105.2 (102.6, 108.3)	0.657
Calcium (mmol/L)	2.2 (2.1, 2.3)	2.1 (2.0, 2.2)	<0.001
White blood cell count (10^9/L)	6.7 (5.7, 8.7)	7.4 (5.8, 9.2)	0.081
Hematocrit (%)	39.1 ± 5.2	37.0 ± 5.5	<0.001
Platelet count (10^9/L)	188.5 (166.0, 239.0)	194.0 (150.0, 265.0)	0.937
Prothrombin time (s)	12.5 (11.5, 13.6)	13.4 (11.9, 14.5)	<0.001
International normalized ratio	1.0 (1.0, 1.1)	1.1 (1.0, 1.2)	<0.001
Activated partial thromboplastin time (s)	29.6 (25.7, 37.1)	33.8 (27.9, 40.9)	<0.001
Fibrinogen (g/L)	3.7 (3.0, 4.5)	3.9 (3.2, 4.6)	0.086
Thrombin time (s)	16.2 (15.2, 17.0)	16.0 (15.2, 16.9)	0.481
D dimer (mg/L)	2.1 (1.2, 5.3)	3.6 (2.1, 7.6)	<0.001
Fibrinogen degradation products (mg/L)	7.1 (4.1, 16.2)	13.1 (6.7, 23.7)	<0.001
Antithrombin (g/L)	83.8 (74.0, 91.0)	79.7 (72.0, 88.0)	0.073
Surgical options			0.164
Femoral head replacement	102 (73.9%)	138 (81.2%)	
Total hip replacement	36 (26.1%)	32 (18.8%)	
Anesthesia method			0.022
Intravertebral anesthesia	111 (80.4%)	116 (68.2%)	
General anaesthesia	27 (19.6%)	54 (31.8%)	
Start time of surgery			0.914
Before 16:00 p.m.	128 (92.8%)	156 (91.8%)	
After 16:00 p.m.	10 (7.2%)	14 (8.2%)	
Operative center rate			0.703
Normal	118 (85.5%)	149 (87.6%)	
Abnormal	20 (14.5%)	21 (12.4%)	
Intraoperative systolic blood pressure			0.102
Normal	56 (40.6%)	86 (50.6%)	
Abnormal	82 (59.4%)	84 (49.4%)	
Intraoperative diastolic blood pressure			0.162
Normal	151(88.8%)	114 (82.6%)	
Abnormal	19 (11.2%)	25 (17.4%)	
Blood loss (mL)	200.0 (100.0, 200.0)	200.0 (100.0, 300.0)	0.044
Duration of surgery(min)	100.0 (90.0, 120.0)	115.0 (90.0, 120.0)	0.117
Total fluid take (mL)	1500.0 (1000.0, 1600.0)	1500.0 (1500.0, 2000.0)	<0.001
Crystalloid_solution (mL)	1000.0 (500.0, 1000.0)	1000.0 (600.0, 1100.0)	0.024
Red blood cell transfusion			0.007
No	127 (92%)	137 (87.4%)	
Yes	11 (8%)	33 (12.6%)	
Intraoperative urine volume(mL)	200.0 (100.0, 400.0)	225.0 (100.0, 400.0)	0.234

### Univariate analysis

Univariate logistic regression analysis was performed to screen for potential factors associated with postoperative hypoalbuminemia. A total of 18 perioperative variables were identified as significantly correlated with the development of postoperative hypoalbuminemia (*p* < 0.05). These variables covered age, preoperative anemia, total protein (TP), hydroxybutyrate dehydrogenase (HBDH), creatine kinase (CKMB), triglyceride (TG), calcium (CA), hematocrit (HCT), prothrombin time (PT), international normalized ratio (INR), activated partial thromboplastin time (APTT), D-dimer, Fibrinogen degradation products (FDP), Anesthesia method, Blood loss, Total fluid take, Crystalloid solution, Red blood cell transfusion (Table 1).

### Multivariate logistic regression analysis

To identify independent risk factors for postoperative hypoalbuminemia, all variables with significant associations in the univariate analysis (*p* < 0.05) were included in a stepwise forward multivariate logistic regression model. The multivariate analysis revealed that the following five variables were independent risk factors for postoperative hypoalbuminemia in elderly patients undergoing hip arthroplasty (Table 2): total fluid intake, TP, HBDH, INR, D-dimer.

**Table 2:** Multivariate logistic regression analysis.

Variables	*β*	S.E.	Wald	*p*-value	*OR*	OR 95% CI
Total fluid intake	1.417	0.487	8.489	0.004	4.124	1.588, 10.708
TP	0.921	0.370	6.205	0.013	2.511	1.217, 5.182
HBDH	1.041	0.362	8.270	0.004	2.833	1.393, 5.760
INR	1.393	0.536	6.758	0.009	4.029	1.409, 11.519
D-dimer	0.857	0.415	4.258	0.039	2.355	1.044, 5.314

### Risk prediction model

**Figure 2:**
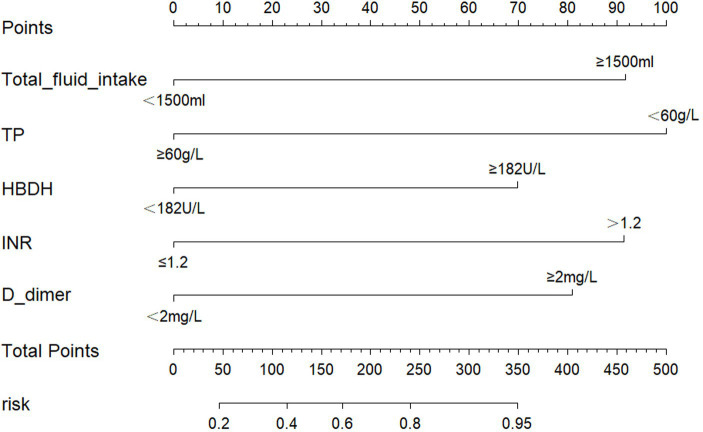
The nomogram prediction model.

**Figure 3:**
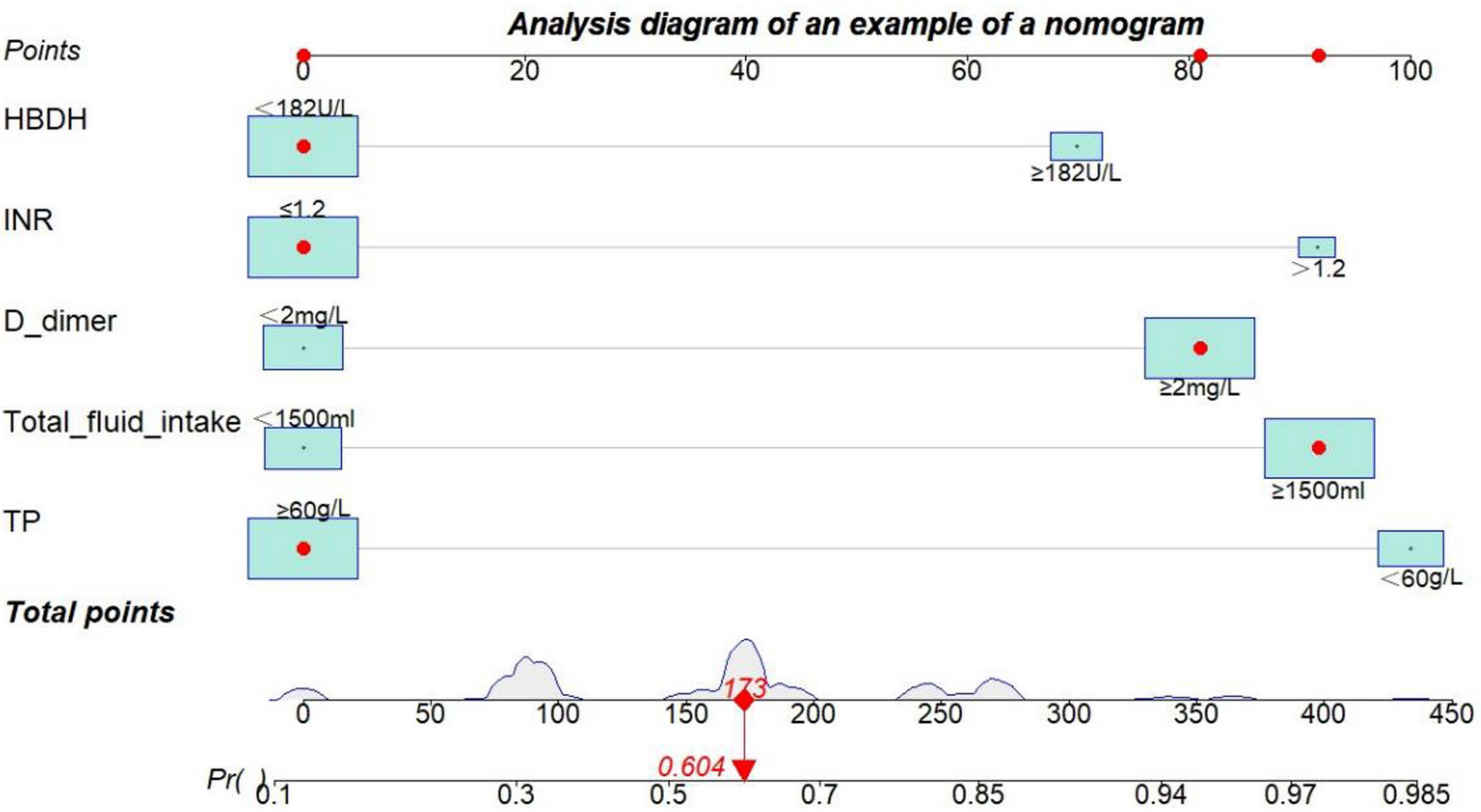
Analysis diagram of an example of a nomogram.

Based on the results of the multivariate stepwise logistic regression analysis, a risk prediction model for postoperative hypoalbuminemia in elderly hip arthroplasty patients was constructed. Predicted probability: *p* = 1/[1 + e^−2.731 + 1.417 × Total fluid intake+0.921 × TP + 1.041 × HBDH+ 1.393 × INR + 0.857 × D-dimer^]. To enhance clinical applicability, a nomogram was generated to visualize the model, with each independent risk factor assigned a corresponding score based on its regression coefficient (Figure 2). The total score of an individual patient—calculated by summing the scores of all applicable factors—could be mapped to the predicted probability of postoperative hypoalbuminemia. A representative example of the nomogram’s application, using a randomly selected patient from the study cohort, is provided to demonstrate its practical use (Figure 3).

**Figure 4:**
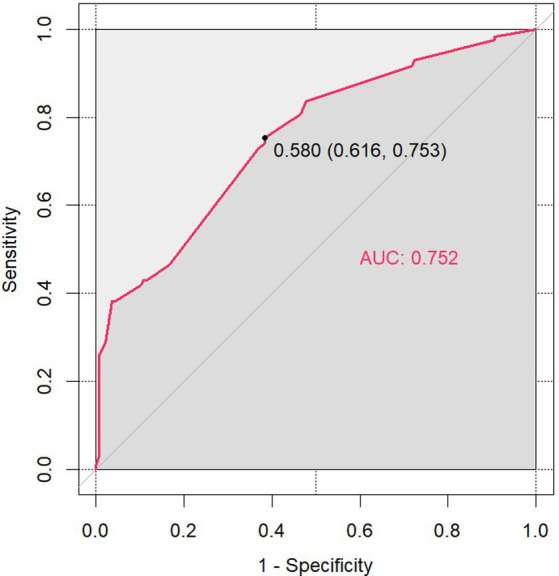
ROC curve and AUC value of the risk prediction model.

Receiver operating characteristic (ROC) curve analysis was performed to evaluate the model’s discriminative ability. The area under the ROC curve (AUC) was 0.752 (95% CI: 0.699–0.805), with a sensitivity of 61.6% and a specificity of 75.3% (Figure 4). Additionally, the concordance index (C-index) of the model was 0.752 (95% CI: 0.699–0.805)—a value consistent with the AUC, confirming moderate-to-good discriminative performance (C-index > 0.7 indicates favorable distinction between patients who do and do not develop the outcome).

**Figure 5:**
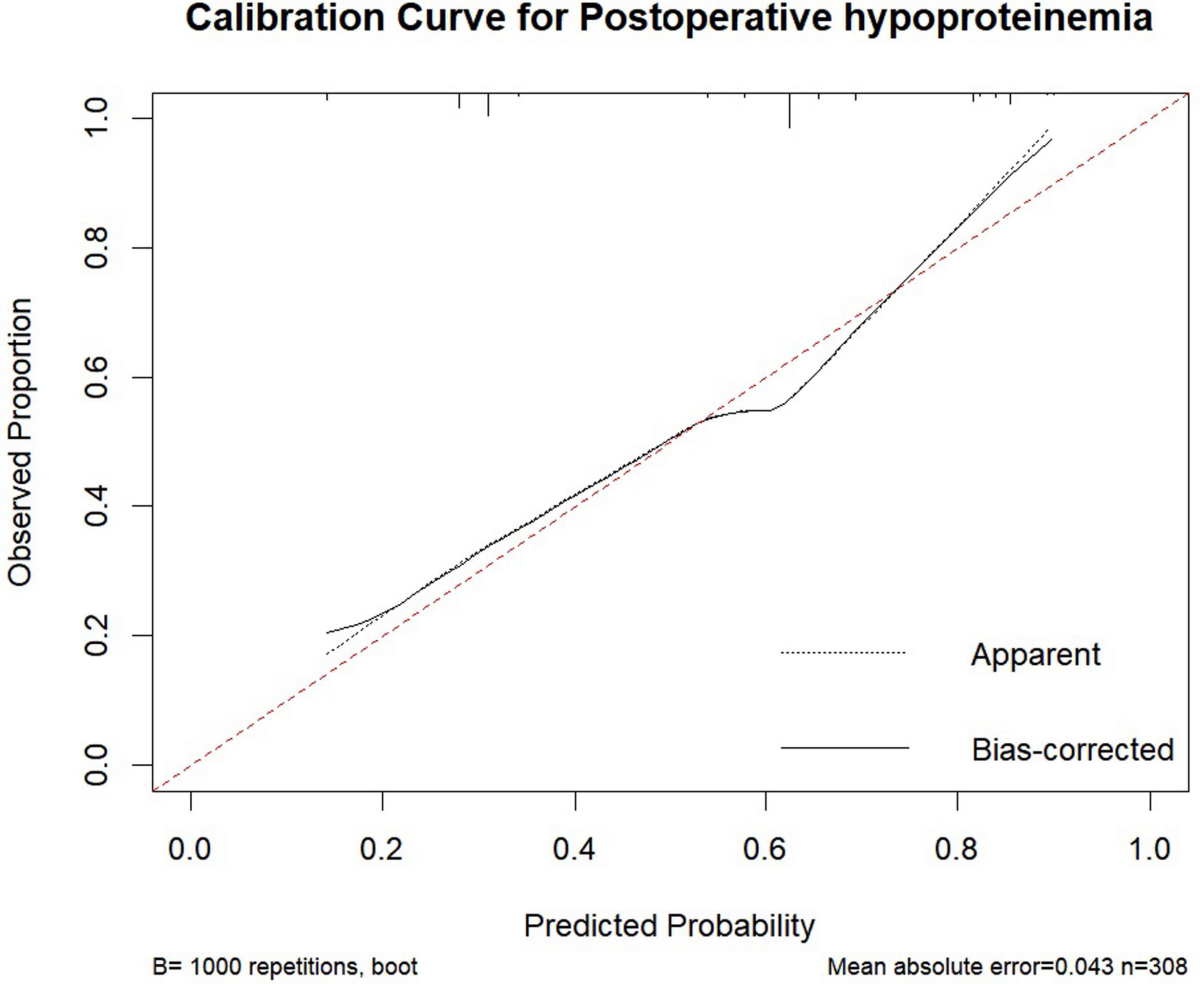
The calibration curve of the risk prediction model.

Calibration of the model was assessed using a calibration curve and the Brier score, alongside the previously reported Hosmer–Lemeshow test. The calibration curve showed close alignment between the predicted probabilities of the nomogram and the observed rates of postoperative hypoalbuminemia (Figure 5). The Hosmer–Lemeshow test yielded *χ*^2^ = 6.997 (*p* = 0.537), further confirming no significant difference between predicted and observed outcomes. The Brier score was 0.355—while slightly higher than the ideal value, it remained within a range indicating acceptable agreement between predicted probabilities and actual event rates (Figure 5).

**Figure 6:**
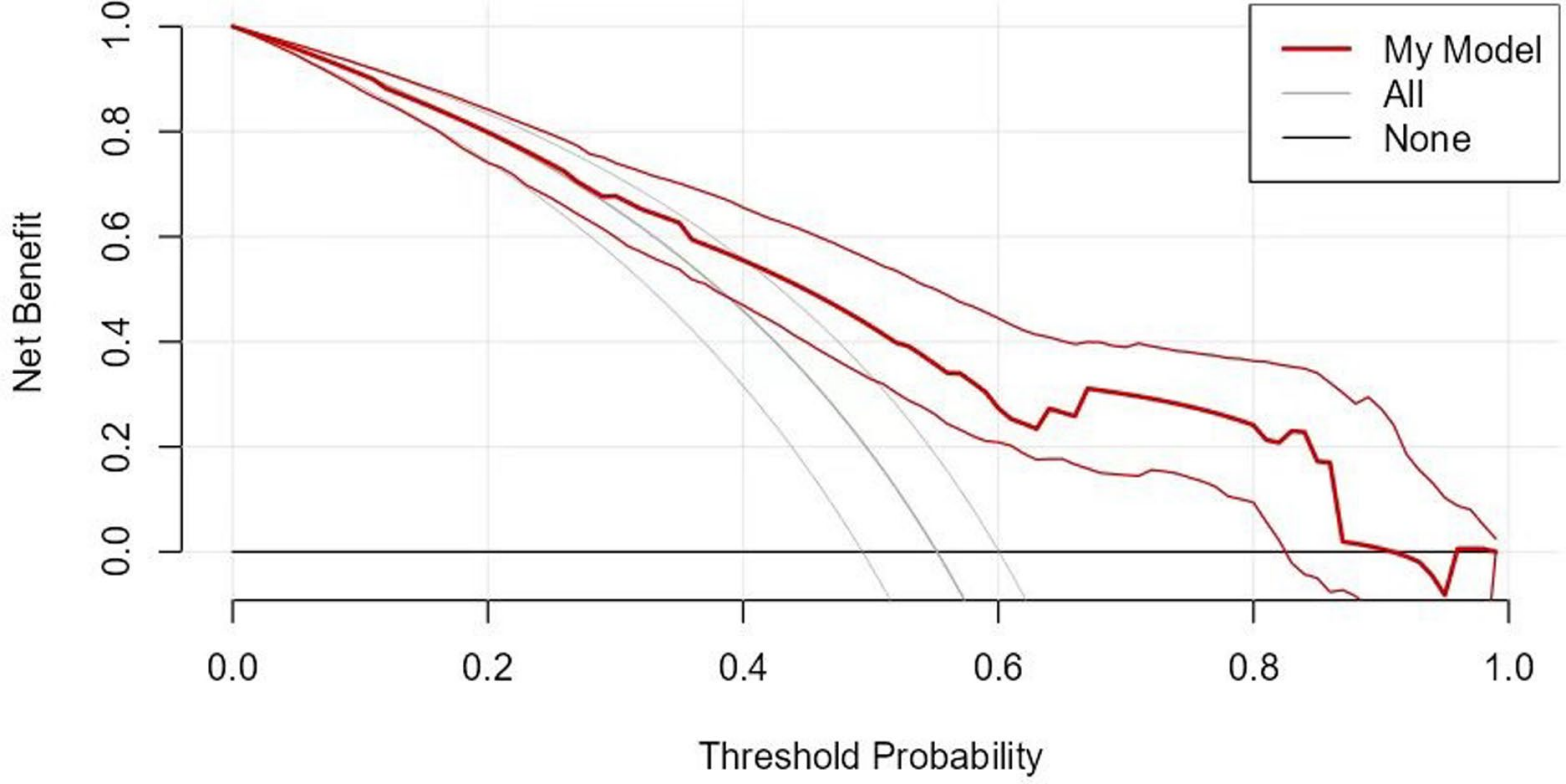
Decision curve analysis of the risk prediction model.

Decision curve analysis (DCA) was conducted to evaluate the model’s clinical utility by estimating the net benefit (NB) across different threshold probabilities of postoperative hypoalbuminemia. The x-axis of the DCA curve represented the threshold probability (Pₜ), and the y-axis represented the net benefit (calculated as the benefit of correctly identifying high-risk patients minus the harm of incorrectly classifying low-risk patients). A patient was classified as “high-risk” if their predicted probability (Pᵢ) exceeded Pₜ. As shown in Figure 6, the model achieved a positive net benefit across a wide range of threshold probabilities (0.01–0.90). This indicated that using the nomogram to guide clinical decisions would result in more clinical benefit than either treating all patients or treating none—confirming the model’s practical value in perioperative care (Figure 6).

### Outcomes

Retrospective analysis was performed to compare postoperative outcomes between the hypoalbuminemia and control groups, using the Mann–Whitney U test for continuous variables and the chi-square test for categorical variables. No statistically significant differences were observed between the two groups in terms of postoperative hospital stay duration, incidence of postoperative acute kidney injury, incidence of postoperative cardiovascular events, or overall mortality (*p* > 0.05 for all). However, several key outcomes showed notable disparities: The incidence of postoperative pneumonia in the hypoalbuminemia group was 18.2% (31/170), significantly higher than that in the control group, which was 10.9% (15/138). The rate of postoperative ICU admission was significantly higher in the hypoalbuminemia group compared to the control group (*p* < 0.05, detailed data in Table 3). Although the difference did not reach statistical significance (*p* = 0.087), the mortality rate was 2.4% (4/170) in the hypoalbuminemia group versus 0% (0/138) in the control group.

**Table 3:** The outcomes of patients with postoperative hypoalbuminemia.

Outcomes	Control group (*n* = 138)	Hypoproteinemia group (*n* = 170)	*p*
Postoperative hospitalstay	7.0 (6.0 to 10.0)	7.0 (5.0 to 11.0)	0.918
Postoperative pneumonia			0.1
No	123 (89.1%)	139 (81.8%)	0.1
Yes	15 (10.9%)	31 (18.2%)	
Postoperative acute kidney injury			0.329
No	134 (97.1%)	160 (94.1%)	
Yes	4 (2.9%)	10 (5.9%)	
Postoperative delirium			0.894
No	128 (92.8%)	157 (92.4%)	
Yes	10 (7.2%)	13 (7.6%)	
Postoperative cardiac complications			0.516
No	133 (96.4%)	160 (94.1%)	
Yes	5 (3.6%)	10 (5.9%)	
Postoperative ICU			<0.001
No	129 (93.5%)	120 (70.6%)	
Yes	9 (6.5%)	50 (29.4%)	
Mortality			0.191
No	138 (100%)	166 (97.6%)	
Yes	0 (0%)	4 (2.4%)	

## Discussion

Previous studies have reported that serum albumin levels decrease by approximately 10–15 g/L within 1 week after major surgery, with capillary leakage being the main contributing factor, which is a key factor disrupting perioperative nutritional homeostasis (7, 12). Hip arthroplasty, a major orthopedic procedure, induces traumatic capillary leak syndrome (endothelial injury and increased vascular permeability), promoting albumin extravasation. Surgical trauma also triggers systemic inflammation, with proinflammatory cytokines suppressing hepatic albumin synthesis, which relies on adequate nutritional substrates (e.g., essential amino acids) (13). Our univariate analysis identified 18 perioperative variables significantly associated with postoperative hypoalbuminemia (*p* < 0.05). Multivariate logistic regression further narrowed these to five independent risk factors: total fluid intake, TP, HBDH, INR, and D-dimer.

Our study identified higher intraoperative total fluid intake as an independent risk factor, as it exacerbates hemodilution and albumin loss. This highlights the value of goal-directed fluid therapy (GDFT) in perioperative nutrition management, which mitigates hemodilution and capillary leakage (7, 14). Notably, the mechanism regulating the intravascular-extravascular albumin ratio remains poorly understood. Elevated postoperative INR (>1.2) and D-dimer (≥2 mg/L) were independent risk factors. INR elevation indicates reduced synthesis of hepatocyte-derived vitamin K-dependent coagulation factors (15). Since the liver is the sole site of albumin synthesis, impaired hepatic function (often linked to preoperative malnutrition in the elderly) concurrently reduces both coagulation factors and albumin. Studies by Violi. F et al. and Aloisio. E et al. have shown that hypoproteinemia is associated with elevated D-dimer levels, which is consistent with our results (16, 17). However, the causal relationship between hypoproteinemia and coagulation factors remains undetermined, and further research is needed to support this hypothesis. Elevated preoperative HBDH, a novel predictor of postoperative hypoalbuminemia in this population, reflects LDH isoenzyme activity in hepatocytes and other cells (18). Its elevation indicates subclinical tissue injury or metabolic dysfunction, which impairs nutritional metabolism and albumin synthesis (19). Two nutrition-related mechanisms may be involved: (1) Inflammation-induced HBDH release via cytokines (e.g., IL-6, TNF-*α*) suppresses hepatic albumin synthesis; (2) Comorbidities (e.g., heart disease) elevate HBDH, reduce hepatic perfusion, and decrease albumin synthesis. Additionally, skeletal muscle injury also increases protein catabolism, depleting amino acid reserves for albumin synthesis. Preoperative TP < 60 g/L, a direct marker of preoperative malnutrition, was an independent risk factor, consistent with findings by Critselis et al. (20). Serum proteins maintain colloid osmotic pressure and provide nutritional support, critical for perioperative recovery. This supports preoperative nutritional assessment and multidisciplinary intervention, which improve nutritional status, reduce postoperative hypoalbuminemia, and lower hospital costs (21).

As shown in Table 3, patients with postoperative hypoalbuminemia suffered a higher burden of adverse short-term outcomes, including significantly elevated rates of postoperative pneumonia, acute kidney injury, and ICU admission. Notably, ICU admission differed drastically between groups (29.4% vs. 6.5%, *p* < 0.001), representing the most clinically meaningful result of this analysis. Unplanned ICU transfer consumes scarce critical care resources and substantially raises overall perioperative medical costs, underscoring the urgent need for preoperative risk screening. Though 30-day mortality was numerically higher in the hypoalbuminemia group (2.4% vs. 0%) without statistical significance (Fisher’s exact test, *p* = 0.131) due to limited death events, this unfavorable prognostic pattern matches Kaya et al., who confirmed low albumin and inflammatory markers jointly predict mortality in elderly hip fracture patients, supporting the clinical utility of our multi-biomarker nomogram for early risk stratification and cost-saving targeted interventions (22).

Beyond infectious, renal and critical care complications shown in Table 3, elderly hip fracture patients are also susceptible to perioperative neurocognitive disorders (PND) such as postoperative delirium, driven by malnutrition and systemic inflammation (23, 24). Prior prospective trials of geriatric arthroplasty patients verified that hypoalbuminemia and elevated proinflammatory cytokines jointly amplify neuroinflammation and cognitive impairment, which may be alleviated via optimized anesthetic and fluid regimens. However, standardized cognitive evaluations were unavailable in our retrospective cohort, precluding subgroup analysis of POD risk.

Our nomogram addresses these gaps by integrating five independently validated risk factors, all of which are routinely measured in clinical practice (e.g., TP, HBDH, INR, D-dimer) or easily recorded (total fluid intake). The model demonstrated moderate-to-good discriminative ability (AUC = 0.752), good calibration (Hosmer–Lemeshow *p* = 0.537, Brier score = 0.155), and favorable clinical utility (positive net benefit across threshold probabilities of 0.01–0.90). Compared with previous risk prediction models for postoperative complications in patients undergoing hip replacement, our nomogram incorporates new independent predictors (HBDH and D - dimer), which have not been included in the prediction models for hypoalbuminemia in elderly patients undergoing hip replacement.

By quantifying individual risk, the nomogram enables clinicians to identify high-risk patients preoperatively and implement targeted interventions: for example, GDFT for patients with expected high fluid intake, nutritional support for those with low preoperative TP, or closer monitoring of coagulation and liver function for patients with elevated INR or D-dimer. By implementing targeted perioperative optimization strategies and applying this nomogram to the perioperative care of elderly patients undergoing hip replacement surgery, unnecessary albumin infusion and postoperative intensive care unit (ICU) admissions can be reduced. This can lower medical costs, improve the efficiency of clinical resource allocation, and simultaneously reduce the incidence of complications related to hypoalbuminemia.

Notably, our cohort excluded patients with preoperative albumin <30 g/L to isolate newly-onset postoperative hypoalbuminemia, which means the current nomogram is only suitable for elderly hip arthroplasty patients with normal or mild preoperative nutritional status. For patients with severe preoperative hypoalbuminemia, independent risk factors and predictive thresholds may differ; future extended research covering severely malnourished subgroups is required to expand the generalizability of this risk prediction tool.

This study has several limitations. First, a relatively small sample size. Second, a relatively limited statistical power. Third, preoperative laboratory indices were only measured on the day of admission (missing longitudinal preoperative data), and postoperative indices were only collected on the day of surgery (omitting dynamic changes in albumin or biomarkers). Fourth, we did not assess other potential predictors, such as preoperative nutritional scores, medication use (e.g., corticosteroids), or postoperative pain management (which may affect catabolism). Fifth, the study covered a 10-year patient enrollment period (2015–2024), during which institutional anesthesia protocols and intraoperative fluid management strategies were gradually updated, and such long-term protocol heterogeneity may bring unmeasured confounding effects to the analysis results. Sixth, we screened over 30 variables and retained 18 significant predictors for multivariate regression. Multiple univariate comparisons may inflate type I error risk, despite stepwise filtering. Future studies should adopt predefined variables or multiple comparison correction to reduce this bias.

Future research should: (1) Conduct multicenter prospective studies with larger sample sizes to validate and refine the nomogram, and explore associations between hypoalbuminemia and long-term outcomes (e.g., 90-day mortality, readmission rates). (2) Incorporate additional variables such as nutritional scores, medication use, and longitudinal laboratory data to improve model accuracy.

## Conclusion

Elderly patients undergoing hip arthroplasty are at high risk of postoperative hypoalbuminemia, a common nutrition-related complication reflecting perioperative nutritional imbalance, with a reported incidence of 55.19% in this study. We identified total fluid intake, TP, HBDH, INR and D-dimer as five independent risk factors for postoperative hypoalbuminemia. We further developed a nomogram- based model for preoperative risk stratification, which demonstrated favorable performance, excellent calibration and a positive clinical utility. The model is practical for guiding targeted clinical nutritional interventions, thereby optimizing patients’ nutritional status and improving their clinical outcomes.

## Data Availability

The raw data supporting the conclusions of this article will be made available by the authors, without undue reservation.
